# The λ pattern on time-RR interval scatter plot of neonatal ambulatory ECG: a marker of transient bradycardia

**DOI:** 10.3389/fcvm.2025.1663243

**Published:** 2025-09-05

**Authors:** Hualian Li, Xin Wei, Fengna Zhu, Fei Zheng, Tingting Yu

**Affiliations:** ^1^ Electrocardiogram Diagnostic Department, Hubei Maternal and Child Health Hospital, Wuhan, China; ^2^ Neonatology Department, Hubei Maternal and Child Health Hospital, Wuhan, China

**Keywords:** AECG, bradycardia, neonate, T-RR scatter plot, escape rhythm

## Abstract

**Background:**

Neonatal bradycardia often triggers transient escape rhythms that challenge clinical diagnosis, with current methods lacking dynamic biomarkers for risk stratification.

**Objective:**

To validate the λ pattern, a heart rate dynamic signature on time-RR interval scatter plot, for distinguishing escape rhythms from transient sinus bradycardia and predicting recovery timelines in neonates.

**Methods:**

Retrospective analysis of 36 neonates (≤28 days) with 24 h electrocardiogram (ECG) monitoring. Holter data identified λ patterns (abrupt ≥20% RR prolongation and >3 s gradual recovery). Reverse-engineering ECG validated rhythm origins. Survival models assessed λ burden-prognosis correlations.

**Results:**

487 λ patterns (15.5 ± 3.2/neonate) were detected: 80.3% escape rhythms, 19.7% sinus bradycardia. High λ burden (≥21/24 h) predicted delayed recovery vs. low burden (≤10/24 h) [HR = 4.22 (95% CI: 1.98–9.01), *p* < 0.0001]. All cases resolved spontaneously within 6 months.

**Conclusion:**

The λ pattern shows promise as a noninvasive biomarker for stratifying neonatal bradycardia and shows potential to guide recovery timeline prediction. Integration of this approach could optimize neonatal arrhythmia management.

## 1 Introduction

Bradycardia represents a relatively common arrhythmia in neonates (1). Neonatal bradycardia is diagnosed when the heart rate falls below 100 beats per minute (BPM), with etiologies broadly categorized into two mechanistic pathways: (1) suppression of normal sinus node activity due to autonomic instability (non-cardiac factors), and (2) intrinsic sinus node dysfunction caused by congenital structural anomalies, reversible perinatal insults like hypoxia, severe infection or metabolic disturbances or sinoatrial conduction block (2, 3).

When bradycardia or sinus arrest occurs, the most frequent compensatory mechanism is escape rhythm (ER), a passive arrhythmia originating from the atrioventricular junction, atrium or ventricle to maintain hemodynamic stability (4). In neonates, junctional ER (JER) emerges as the predominant cardiac rhythm disturbance, typically demonstrating a characteristic rate range of 80–120 BPM. This rhythm exhibits electrocardiographic features including narrow QRS complexes (<80 ms duration) with regular RR intervals. While JER may transiently reflect physiological sinus node immaturity, its persistence often signals underlying pathology requiring clinical vigilance (5).

Conventional 12 lead ECG, limited by brief recording periods (seconds to minutes), frequently fails to capture paroxysmal JER episodes (6). This modality cannot assess circadian rhythm variations or transient sinus node junctional pacemaker transitions. Ambulatory ECG (AECG) monitoring resolves these limitations through extended continuous recording (7, 8). Modern AECG software further enhances diagnostic precision through analytical tools including t-RR scatter plot, RR interval histograms, and heart rate variability analysis (9). The t-RR scatter plots were constructed by plotting RR intervals (the duration between consecutive heartbeats) on the vertical axis against corresponding time points on the horizontal axis, forming a sequential scatter plot representation of cardiac rhythm dynamics (10, 11). The λ pattern, a novel electrophysiological signature observed on t-RR scatter plots, reflects dynamic interactions between sinus node suppression and compensatory escape rhythms.

Our preliminary investigations revealed a distinctive λ shaped signature on t-RR scatter plots in neonates with bradycardia, reflecting dynamic interplay between suppressed sinus node activity and the emergence of dominant escape rhythms from subsidiary pacemakers. In this study, we integrated t-RR plot analysis with reverse-engineering techniques. This approach enables targeted retrieval and quantification of ECG segments that correspond to specific scatter plot regions, particularly characteristic λ patterns. Such integration facilitates rapid and accurate clinical diagnosis. We identified the *λ* pattern in bradycardic neonates, defined by abrupt heart rate reduction (manifested as RR interval prolongation) followed by gradual rhythm normalization (sinus node recovery), as a consistent electrophysiological signature across atrial ER (AER), JER, and ventricular ER (VER) subtypes. Subsequently, we conducted comprehensive analysis of clinical characteristics and prognostic outcomes, establishing predictive value guiding clinical management. By bridging transient arrhythmia phenomena with actionable clinical insights, this work transforms λ pattern analysis from an observational curiosity into an effective diagnostic-prognostic tool in neonatal cardiology.

## 2 Materials and methods

### 2.1 Population

This retrospective cohort study analyzed AECG recordings from neonates (≤28 days old) admitted to a tertiary neonatal intensive care unit (NICU) between January 2018 and December 2022. Inclusion criteria comprised: (1) ≥24 h AECG monitoring for suspected bradycardia or sinus arrest, and (2) availability of raw rhythm data for t-RR scatter plot generation. Screening identified 52 eligible neonates, with 16 excluded due to insufficient ECG data (*n* = 9) or monitoring <24 h (*n* = 7), leaving 36 for analysis. AECG recordings were initiated within 48 h of NICU admission. At the time of recording, all neonates were hemodynamically stable (defined as no requirement for vasoactive/inotropic agents). As detailed in Results 3.1, common indications included prematurity-related apnea, suspected infection, and metabolic disorders. No patients received autonomic modulators (e.g., atropine, theophylline) during monitoring. Retrospective consent was obtained via structured telephone interviews with legal guardians using IRB-approved scripts. All participants provided verbal consent documented in medical records, with written confirmation mailed.

### 2.2 T-RR plot and reverse-engineering

AECG recordings were obtained using Holter monitors (CT-082, Baihui Company Ltd. Hangzhou, China) and t-RR scatter plots were generated using Holter analysis software (version1.2, Baihui Company Ltd. Hangzhou, China), with each point representing an RR interval plotted against time (*x*-axis: elapsed time in hours; *y*-axis: RR interval). Baseline was defined as the mean RR interval during stable, non-bradycardic periods. The *λ* pattern was defined as a triangular cluster of points showing abrupt RR interval prolongation [≥20% above baseline (12)] indicating heart rate deceleration followed by gradual gradual RR interval shortening indicating rhythm recovery [>3 s: based on sinus node recovery time thresholds (13)]. The λ patterns were reverse-engineered to raw ECG segments through this workflow: Upon identifying λ coordinates within t-RR scatter plots using Holter analysis software, synchronized playback functionality automatically extracted corresponding ECG segments containing QRS complexes. Two board-certified electrophysiologists subsequently performed blinded, independent evaluation of these waveform segments using dual-monitor verification. Cardiac rhythm classification was ultimately determined through consensus adjudication applying established electrophysiological criteria.

### 2.3 Statistical analysis

Statistical analysis was performed using SPSS 26.0. Numerical data were expressed as mean ± SD, and categorical data were expressed as percentages. A *p*-value <0.05 was considered statistically significant.

## 3 Results

### 3.1 Cohort characteristic

The cohort comprised 36 neonates (male: 58.3%) with mean gestational age 35.4 ± 3.8 weeks (range: 28–41 weeks) and birth weight 2.8 ± 0.7 kg. Critical comorbidities included: Respiratory support: CPAP (*n* = 11, 30.6%), mechanical ventilation (*n* = 5, 13.9%). Metabolic disturbances: hypoglycemia (*n* = 7, 19.4%), hypocalcemia (*n* = 3, 8.3%). Hemodynamic status: 31 (86.1%) were asymptomatic; 5 (13.9%) had feeding intolerance. No patients required vasoactive agents.

### 3.2 Rhythm characterization

**Figure 1:**
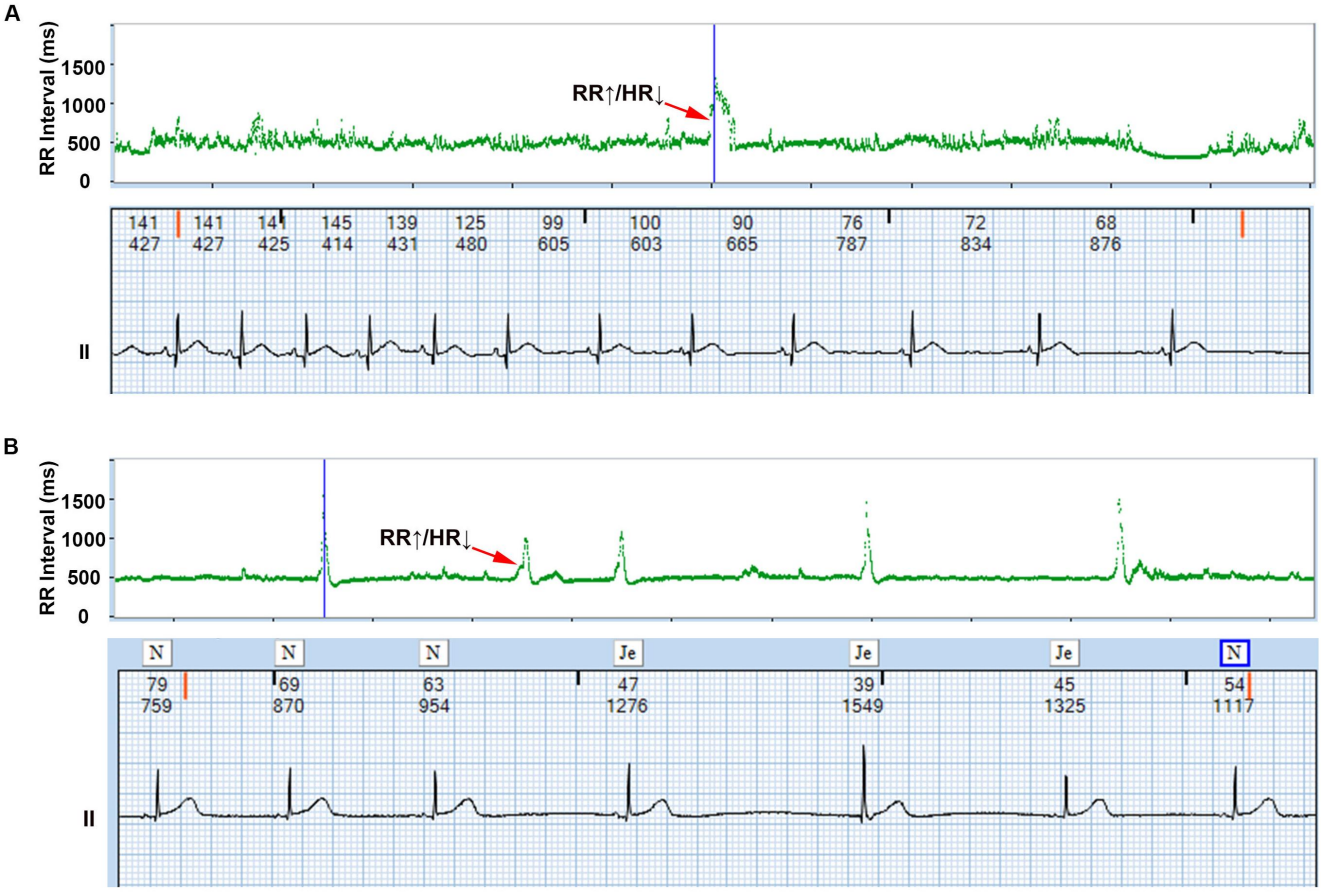
λ pattern of neonatal bradycardia. The upper section displays the λ pattern on the T-RR scatter plot, while the lower section shows the detailed electrocardiogram corresponding to the λ pattern obtained through reverse-engineering technology, and the numbers above the ECG represent the instantaneous heart rate and the RR interval, respectively. Representative examples of *λ* patterns from study cohort: **(A)** Transient sinus bradycardia (Patient #12); **(B)** Junctional escape rhythm (Patient #27). ↑RR interval = ↓Heart rate (Bradycardia), ↓RR interval = ↑Heart rate (Recovery).

The AECG showed that all infants exhibited transient bradycardia, with a mean heart rate of 123 ± 30 BPM and a nadir heart rate of 55 BPM. T-RR scatter plot analysis identified 487 λ patterns (mean 15.5 ± 3.2 per neonate). Reverse-engineering confirmed 391 λ patterns (80.3%) as ERs (predominantly junctional origin) and 96 (19.7%) as transient sinus bradycardia (Figure 1).

### 3.3 Clinical outcome

**Figure 2:**
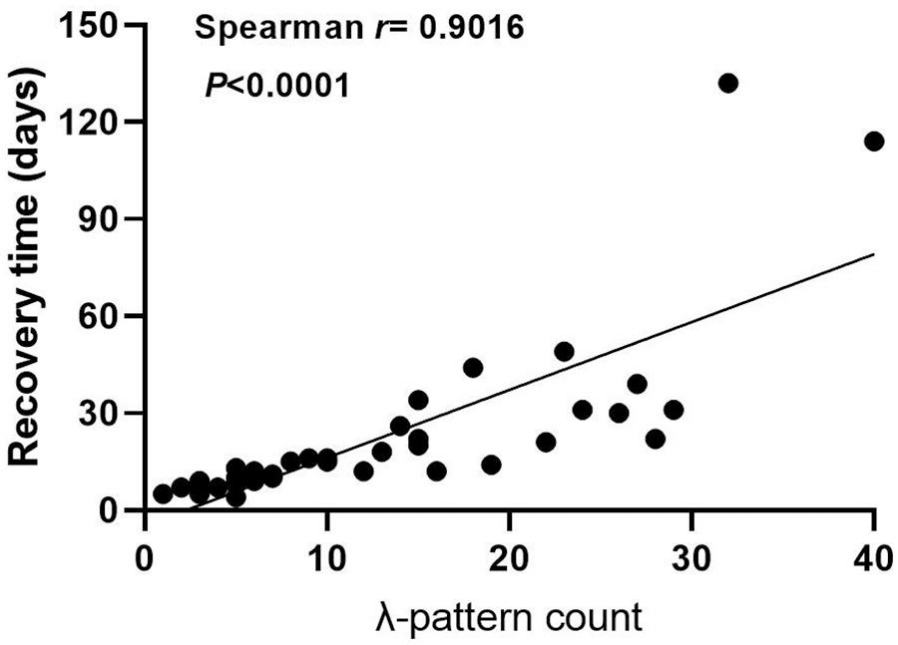
Association between λ pattern counts and rhythm normalization time. Each point represents an individual patient.

Among 36 neonates with confirmed bradycardia, 8 cases (22.2%) exhibited I to II degree atrioventricular block or prolongation of the QT interval (I degree: 3, II degree: 2, prolonged QT interval: 3), all resolving spontaneously within 6 months post-discharge (median recovery time 98 days). We constructed a scatter plot with λ pattern counts as the *x*-axis (using the maximum count from multiple Holter recordings when applicable) and recovery duration as the *y*-axis. The analysis revealed a significant positive correlation (Figure 2).

**Figure 3:**
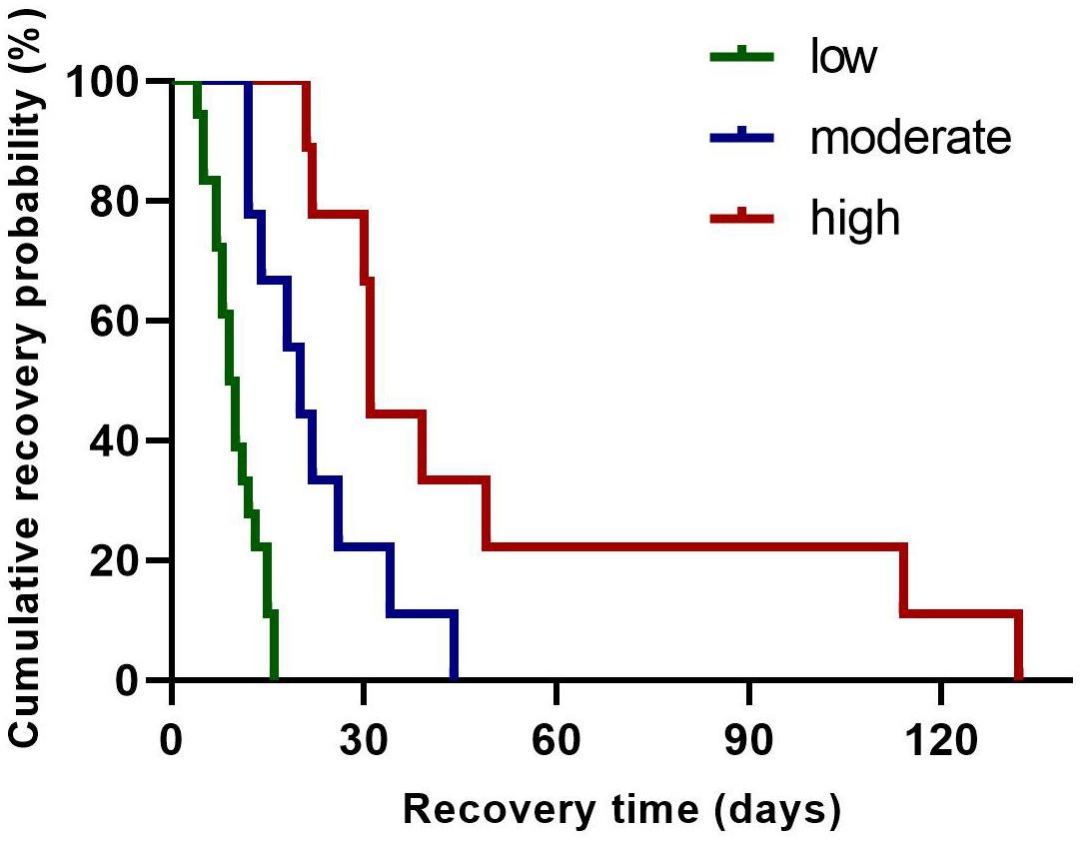
Kaplan–Meier survival analysis of rhythm normalization time across λ pattern groups. The survival curve represents the probability of patients remaining in arrhythmia over time. Groups are stratified by λ pattern: low (green line), moderate (blue line), and high (red line).

Following tertile-based stratification of neonates into λ-pattern burden groups [low (≤10 episodes/24 h), moderate (10–20 episodes/24 h), and high (≥21 episodes/24 h)], Kaplan–Meier analysis demonstrated a significant association between *λ*-pattern burden and delayed rhythm normalization (log-rank *χ*^2^ = 22.39, *p* < 0.0001). Neonates in the high-burden group (≥21 episodes/24 h) showed a 4.22-fold increased risk of delayed recovery compared to those in the low-burden group (≤10 episodes/24 h; Figure 3). Median recovery time differed substantially between groups: 62 days (IQR 45–78) in the low-burden cohort vs. 121 days (IQR 89–152) in the high-burden cohort.

## 4 Discussion

Neonatal bradycardia, defined as a heart rate <100 bpm, primarily stems from autonomic nervous system immaturity during the perinatal period (2, 14). Characterized by heightened vagal tone, this condition manifests through clinically observable triggers including apneic episodes, feeding, and defecation (15). While most instances exhibit benign self-limiting behavior, recurrent bradycardic events warrant thorough investigation for potential systemic stressors such as infection, sepsis or hypoxic-ischemic injury (5, 16). ERs act as critical physiological safeguards against severe bradyarrhythmias (17). Our study introduces λ-wave morphology, derived from T-RR scatter plot analysis, as both a diagnostic biomarker and dynamic tracker of sinus node dysfunction. This triphasic electrophysiological signature enables real-time assessment of sinus node recovery capacity.

The *λ* pattern demonstrates a distinctive biphasic morphology characterized by a sharp heart rate deceleration phase followed by gradual acceleration. This pattern physiologically reflects the dynamic interaction between primary and subsidiary cardiac pacemakers. Reverse ECG mapping analysis confirms that this pattern signifies either escape rhythms or transient sinus bradycardia, with the initial acceleration phase corresponding to either the emergence of ERs (atrial, junctional, or ventricular) during sinus node dysfunction or transient sinus bradycardia triggered by autonomic fluctuations. The subsequent deceleration phase marks sinus node functional recovery through overdrive suppression of ectopic foci, a process dynamically regulated by autonomic balance (18). This mechanistic framework aligns with established electrophysiological principles while introducing a novel noninvasive diagnostic parameter—the λ pattern. The integration of λ pattern kinetics with ECG reverse-mapping technology enables rapid, intuitive bradycardia identification, representing a paradigm shift in neonatal cardiac monitoring. Clinically, this advancement may improve prediction accuracy for pacemaker requirements while reducing unnecessary interventions in transient autonomic dysfunction cases.

Furthermore, our retrospective cohort analysis revealed a correlation between λ pattern burden and both arrhythmia severity (*p* < 0.01) and recovery duration (*p* = 0.003), potentially positioning it as a potential prognostic biomarker. Quantitative assessment of λ pattern dynamics may provide an innovative clinical framework for predicting recovery trajectories, bridging critical diagnostic gaps in detecting evolving conduction abnormalities, inflammatory cascades, and autonomic instability. This methodology could enhance diagnostic precision and conservative management efficacy in neonates, enabling personalized arrhythmia management strategies that optimize both therapeutic precision and healthcare resource allocation.

This retrospective observational study has several limitations. Given the moderate sample size (*n* = 36), our findings require validation in larger cohorts. While *λ*-pattern burden suggests prognostic utility, clinical applications should be cautious pending multicenter confirmation. The design inherently prevents establishing causal relationships between λ pattern characteristics and clinical outcomes, while the ≥24 h AECG monitoring inclusion criterion introduces selection bias by potentially excluding mild/asymptomatic cases. Single-center enrollment limits generalizability to broader populations, and methodological constraints include semi-automated λ pattern identification with inherent subjectivity, compounded by incomplete therapeutic documentation that hindered precise physiology treatment correlation. Universal standardized care in our cohort precluded assessment of λ specific therapeutic effects, and the moderate sample size requires external validation in larger cohorts. These limitations highlight the need for prospective multicenter studies employing protocolized monitoring and detailed therapeutic records to verify clinical applications.

## Data Availability

The original contributions presented in the study are included in the article/Supplementary Material, further inquiries can be directed to the corresponding author.
